# Acceptability and uptake of COVID-19 vaccines among pregnant and lactating women seeking services at a tertiary public hospital in Kampala, Uganda

**DOI:** 10.1186/s12884-026-09708-8

**Published:** 2026-07-25

**Authors:** Elizabeth B. Katana, Eva Agnes Laker Odongpiny, Brenda N. Simbwa, Edirisa J. Nsubuga, Josephine Namayanja, Richard Muhindo, Daniel Kiiza, Barbara Castelnuovo, Alex R. Ario, Catriona Waitt

**Affiliations:** 1 Uganda National Institute of Public Health, Ministry of Health, Kampala, Uganda; 2 Infectious Diseases Institute, College of Health Sciences, Makerere University, Kampala, Uganda; 3 Uganda National Expanded Programme on Immunization, Ministry of Health, Kampala, Uganda; 4 National Public Health Emergency Operations Centre, Ministry of Health, Kampala, Uganda; 5 Department of Women’s and Children’s Health, University of Liverpool, Liverpool, UK

**Keywords:** Pregnant women, Lactating women, Vaccination, Vaccine acceptability, Vaccine uptake, COVID-19 vaccines, Maternal vaccination, Uganda

## Abstract

**Background:**

Globally, several vaccines, including COVID-19 vaccines, have been routinely recommended during pregnancy and lactation. However, data on COVID-19 vaccine acceptability and uptake among pregnant and lactating women are limited in Sub-Saharan Africa. While COVID-19 is no longer a public health emergency it is important to take stock of lessons learnt to prepare for future health threats including those that disproportionately affect pregnant/lactating women. We aimed to assess acceptability and uptake of COVID-19 vaccines among pregnant and lactating women in Uganda.

**Methods:**

This was a cross-sectional study conducted among 424 pregnant and lactating women, who were seeking maternal and child health care at Kawempe National Referral Hospital in Kampala conducted between May and July 2023. We obtained data on vaccine acceptability defined as willingness to accept vaccines using interviewer-administered questionnaires. In addition, we assessed vaccination status. Factors associated with COVID-19 vaccine acceptability and uptake were evaluated using modified Poisson regression.

**Results:**

The mean age of the respondents was 26.9 years (SD = 5.7), ranging from 14 to 45 years. Among 424 respondents, 51.6% had received at least one dose of COVID-19 vaccine, 94.1% had received tetanus toxoid vaccine, and 48.4% regardless of their vaccination status, were willing to receive COVID-19 vaccines. Only 5/212(2.4%) had received COVID-19 vaccines while lactating with none having been received during pregnancy. Factors associated with COVID-19 vaccine uptake included history of testing for COVID-19 (aPR = 1.92, CI:1.46–2.54) and having a vaccinated household member (aPR = 1.34, CI:1.03–1.84). COVID-19 vaccine willingness was significantly associated with being a household head (aPR = 2.2, CI:1.12–4.27) and having a vaccinated household member (aPR = 1.33, CI:1.04–1.76).

**Conclusion:**

COVID-19 vaccine acceptance among pregnant and lactating women remains suboptimal despite high acceptance of other maternal vaccines. These findings highlight the need for targeted interventions to improve the acceptance and uptake of COVID-19 vaccines, during pregnancy and lactation. Strengthening risk communication, addressing safety concerns, and leveraging trusted sources of information may enhance vaccine confidence and improve uptake during pregnancy and lactation.

**Supplementary Information:**

The online version contains supplementary material available at https://doi.org/10.1186/s12884-026-09708-8.

## Introduction

Pregnant and lactating women represent a population with unique healthcare needs, particularly concerning the prevention of infectious diseases through vaccination. Vaccination during pregnancy has the potential to confer protection not only to the pregnant woman herself but also to her newborn child, offering a crucial opportunity to safeguard maternal and infant health thus reducing infectious disease-related morbidity and mortality in pregnant women and their infants [1].

Certain vaccines are safe and recommended for women before, during, and after pregnancy to help keep them and their babies healthy [2]. The World Health Organization (WHO) recommends maternal vaccination against tetanus toxoid, pertussis, and influenza, and also recommends COVID-19 vaccination during pregnancy and lactation. Additional vaccines, such as pneumococcal vaccines, may be recommended in specific circumstances [2]. In Uganda, tetanus toxoid (TT) vaccination is routinely recommended for women of reproductive age and pregnant women as part of maternal and neonatal tetanus elimination efforts. Regardless of childhood vaccination history, women aged 15–49 years are offered a schedule of five tetanus vaccine doses during their reproductive years, including doses administered during pregnancy. In contrast, COVID-19 vaccination during pregnancy and lactation was introduced more recently and was not integrated into established maternal immunization programs. Differences in the maturity of these vaccination programs may influence vaccine awareness, trust, and uptake among pregnant and lactating women. However, despite the established benefits of COVID-19 vaccination in this population, uptake and acceptability of COVID-19 vaccines among pregnant and lactating women remain variable across different settings.

Studies have shown that pregnant women with COVID-19 are at a higher risk of developing severe disease and are more likely to suffer adverse outcomes including mortality, preterm births, and preeclampsia when compared with other women of reproductive age [3, 4]. At the start of the COVID-19 pandemic in 2020, most deaths were among older persons and those with co-morbidities, but in 2021, the global increase in COVID-19 deaths among pregnant women became a concern [4].

Based on phase III clinical trials, several vaccines including Pfizer-BioNTech, Moderna and Oxford-AstraZeneca, were proved to be highly efficacious in preventing symptomatic COVID-19 infection in the general population excluding pregnant or lactating women during 2020–2021 [5]. Safety was a main concern for not including pregnant and lactating women in the COVID-19 clinical trials during 2020–2021. By mid-2022, all pregnant women after their first trimester and those who are breastfeeding were eligible for COVID-19 vaccination, as accumulating real-world evidence by April 2021 demonstrated no increased risk of adverse maternal and neonatal outcomes, leading to clear recommendations for vaccination during pregnancy as the risk-benefit ratio changed with more information [6, 7].

In Uganda, there is a lack of comprehensive data and studies on the uptake and acceptability of COVID-19 vaccines among pregnant and lactating women. To address these knowledge gaps, we conducted a study to assess the acceptability and uptake of COVID-19 vaccines among pregnant and lactating women visiting a tertiary public hospital in Kampala between May and July 2023.

Understanding uptake and acceptability to be vaccinated against COVID-19 among pregnant and lactating women will establish what proportion could intentionally not receive the vaccine; this is a potential threat to increased poor disease outcomes. We also compared uptake between COVID-19 vaccine and other routine vaccines to explore potential disease-specific differences in uptake. By exploring factors influencing vaccine decision-making and identifying barriers to COVID-19 vaccination uptake, our study aims to inform targeted interventions and strategies to improve COVID-19 vaccination coverage among this population. This information will also provide insights into plans for future pandemics especially in planning for those that disproportionately result in poor outcomes for pregnant women.

## Methods

### Study design and setting

We conducted a cross-sectional study among pregnant and lactating women visiting Kawempe national referral hospital (KNRH). The study was conducted between May and July 2023. KNRH is specialized in the provision of maternal and neonatal care services and serves as a teaching hospital for Makerere University College of Health Sciences in Kampala, Uganda. It has a 170-bed capacity and offers services to all patients referred from all regions of the country with an average of 57 births daily [8].

We enrolled 212 pregnant and 212 lactating women through a systematic sampling approach. Pregnant women were enrolled at the antenatal unit and lactating women from the postnatal unit. Pregnant and lactating adolescents aged below 18 years were included because adolescent pregnancy remains common in Uganda. The 2022 Uganda Demographic and Health Survey reported that 23.5% of girls aged 15–19 years had initiated childbearing, including 18.4% who had already had a live birth and 5.1% who were pregnant with their first child [9]. These adolescents routinely access maternal and child health services and are eligible for maternal vaccination programs. Their inclusion ensured that the study population was representative of pregnant and lactating women receiving care at the study site.

### Data collection procedure and study questionnaire

The data were collected between May and July 2023 using a structured questionnaire based on survey questions designed to assess determinants of vaccine hesitancy by the SAGE Working group on vaccine hesitancy [10]. These questions were adapted to suit the setting (Ugandan population), respondents (pregnant and lactating women), the disease (COVID-19) and the study objectives. Data were collected electronically using Kobo Toolbox, an open-source platform for mobile data collection and management [11]. Trained research assistants obtained clinical data from the patient’s files and hospital registers from participants who gave informed consent. Participants were then interviewed to obtain information on demographics and other determinants of vaccine hesitancy. Data collection tools were pretested before deployment in 10 participants.

### Study variables

Socio-demographic characteristics included age, sex, education, tribe, religion, marital status, employment, residence, occupation, biological children, household head, household size, health insurance and income.

Clinical factors included pregnancy and lactation related such as gravidity, parity, chronic illness related including type and duration, duration of lactation or pregnancy (weeks of amenorrhea), twin or multiple pregnancy and number of pregnancies since 2020.

COVID-19 disease-related factors included history of testing, confirmed positive test, disease severity, hospitalization, and history of COVID-19 cases or deaths in the household. For COVID-19 vaccination status, the participants were asked about the doses, type of vaccine and when they received the vaccine.

Participants were asked about their willingness to receive the COVID-19 vaccine or additional doses for those who had been vaccinated and if any of their household members were vaccinated against COVID-19. They were also asked about previous uptake since 2020 and their willingness to receive other vaccines, including Tetanus Toxoid (TT) vaccines, which are which are routinely recommended for women of reproductive age and during pregnancy in Uganda. Health system factors included distance from the home to health facility, health education program on vaccination and receiving any information on COVID-19 vaccination from health facilities. Other determinants based on SAGE questionnaire included contextual influences (arising due to historic, socio-cultural, environmental, health system/institutional, economic or political factors), individual and group influences (arising from personal perception of the vaccine or influences of the social/peer environment), and vaccine/vaccination (specific issues directly related to vaccine or vaccination). The SAGE questionnaire is a structured questionnaire based on survey questions designed to assess determinants of vaccine hesitancy by the SAGE Working group on vaccine hesitancy.

Two outcome variables were considered. (1) level of uptake of COVID-19 vaccine (received at least one dose), and (2) acceptability/willingness to receive the COVID-19 vaccine. All the two outcome variables were considered on a binary scale.

### Statistical analysis

All study variables were summarized in forms of frequencies and percentages for categorical variables and means or medians for the continuous variables. The open-ended questions (in free text format) were analysed with a content analysis approach. Modified Poisson regression analysis was used to determine the factors associated with (i) the level of uptake of the COVID-19 vaccine, and (ii) willingness to receive the COVID-19 vaccine. The Prevalence Ratio (PR) was used as the measure of association and p values of less than 0.05 for statistical significance. Bivariate and multivariable analyses were performed. Variables with p-values less than 0.20 at the bivariate analysis stage were considered for inclusion in the multivariable models. The final adjusted models were developed using a stepwise model-building approach, and only variables that remained statistically significant at *p* < 0.05 were retained in the final models. The final adjusted models were checked for goodness of fit. STATA 15 was used for all the analyses.

## Results

### Socio-demographic characteristics of the study participants

Among 424 study participants (212-pregnant and 212-lactating mothers), with mean age of 26.9, ranging from 14 to 45 years, 383 (90.6%) were married/cohabiting, 246 (58%) had achieved secondary level education, 41(9.7%) were heads of their households, 355 (83.7%) had 1–5 live occupants in their households, and 142 (33.5%) had a job, business or were working somewhere. Other participant characteristics are summarized in Table 1.

**Table 1:** Socio-demographic characteristics of the study participants

Variable/Question	Frequency (*n* = 424)	Percentage
Age categories (years)		
≤ 26	218	51.4
> 26	206	48.6
District of residence		
Kampala	179	42.2
Wakiso	220	51.9
Mukono	7	1.7
Others (Kayunga, Kiboga, Luwero, Masaka, Mityana, Mpigi, and Nakasongola)	18	4.2
Distance from home to the nearest public health facility that offers COVID-19 vaccination		
≤ 5 km	185	43.6
> 5 km	239	56.4
Tribe		
Acholi	6	1.4
Alur	4	0.9
Baganda	246	58.0
Bagisu	8	1.9
Bakiga	9	2.1
Banyankore	66	15.6
Banyole	4	0.9
Banyoro	9	2.1
Basoga	25	5.9
Batoro	6	1.4
Lango	2	0.5
Lugbara	2	0.5
Others (Bafumbira, Bakonjo, Bamba, Banyarwanda, Banubi, Basamia, Banyoro, Congolose, Japhadola, Kakwa, Sudanese)	37	8.7
Marital status		
Married/cohabiting	383	90.3
Separated/widowed/divorced	7	1.7
Single/never married	34	8.0
Highest Education level achieved		
None	5	1.2
Primary	110	25.9
Secondary	246	58.0
Tertiary/vocational	34	8.0
University	29	6.8
Religion		
Adventist	10	2.4
Anglican	68	16.0
born-again	95	22.4
Catholic	154	36.3
Muslim	96	22.6
Faith of Unity	1	0.2
House hold head		
No	383	90.3
Yes	41	9.7
Number of biological children (Alive only)		
0	64	15.1
1–2	220	51.9
3–5	124	29.2
≥ 6	16	3.8
House hold size (number of live in occupants)		
1–5	355	83.7
≥ 6	69	16.3
Employment status of respondent (Has a job or business or works somewhere)		
No	282	66.5
Yes	142	33.5
Type/nature of occupation, *n* = 142		
Business owner/operator/attendant	80	56.3
Teacher	17	12
Sales and service (vendor, sales agent, waitress)	18	12.7
Skilled trades (tailor, chef)	10	7
Health worker (nurse, midwife)	4	2.8
Office/administration/finance	9	6.3
Other occupations*	4	2.8
Monthly income in USD, *n* = 112		
> 50	56	50.0
≤ 50	56	50.0
Heard about health insurance		
No	323	76.2
Yes	101	23.8
Had any health insurance since 2020, *n* = 101		
No	97	96.0
Yes	4	4.0
If yes, details of insurance plan, *n* = 4		
Group	0	0
Individual	3	75.0
Work	1	25.0

*Other occupations included casual labour, farming, cleaners/housekeepers, and security personnel

### Clinical factors (pregnancy or lactation related) and COVID-19 disease related factors among study participants

Among 212 pregnant women, 101(47.6%) had a history of 1–2 pregnancies regardless of the outcomes, and 128 (60.4%) were 7–9 months pregnant. Among 212 lactating women, 185(87.3%) were at < 2 months of post child delivery. Among all 424 study respondents, 34 (8.0%) had a chronic illness; of these 10 (29.4%) had gestational hypertension, 7 (20.6%) Asthma, and 5 (14.7%) HIV/AIDS.

Only 130(30.7%) participants had ever tested for COVID-19, 10 (7.7%) had a confirmed positive test. Of these, 4/10 (40.0%) had a history of hospitalisation with COVID-19. Only 21 (4.1%) reported having household members who had ever tested positive for COVID-19 and 5 (1.2%) reported having had household deaths due to COVID-19. Overall, 220(51.9%) reported having household members who had been vaccinated against COVID-19. Other clinical and COVID-19 disease related factors are summarized in Table 2.

**Table 2:** Clinical factors (pregnancy or lactation related) and COVID-19 disease related factors among study participants

Characteristic, *n* = 424	Frequency	Percentage
Gravidity†, *n* = 212		
1–2	101	47.6
3–4	80	37.7
5–6	25	11.8
≥ 7	6	2.8
Parity		
0	56	13.2
1–3	298	70.3
4–5	53	12.5
≥ 6	17	4.0
How many pregnancies between January 2020 and now? including miscarriages		
1	340	80.2
2	66	15.6
≥ 3	18	4.2
Duration of current pregnancy (months of amenorrhea)†, *n* = 212		
1–3 months	6	2.8
4–6 months	78	36.8
7–9 months	128	60.4
Twin or multiple pregnancy†, *n* = 212		
No	202	95.3
Yes	10	4.7
Duration of current lactation (months post-delivery)‡, *n* = 212		
< 2 months	185	87.3
2–4 months	5	2.3
> 4–6 months	22	10.4
Chronic illness(es)		
No	390	92.0
Yes	34	8.0
Type of chronic illness§, *n* = 34		
Chronic Hypertension	5	14.7
Chronic Diabetes Mellitus	1	2.9
HIV/AIDS	5	14.7
Asthma	7	20.6
Hepatitis B	2	5.9
Allergies	2	5.9
Gestational diabetes	2	5.9
Gestational hypertension	10	29.4
Year of chronic illness diagnosis¶, *n* = 27		
2020–2023	8	29.6
2017–2019	8	29.6
2010–2016	4	14.8
< 2010	7	25.9
COVID-19 disease related		
Ever tested for COVID-19		
No	294	69.3
Yes	130	30.7
History of a confirmed positive COVID-19 test, *n* = 130		
No	120	92.3
Yes	10	7.7
History of hospitalisation with COVID-19**, *n* = 10		
Home based care	4	40.0
No	2	20.0
Yes	4	40.0
Disease severity among participants with confirmed COVID-19 infection**, *n* = 10		
Asymptomatic/mild	1	10.0
Moderate disease	8	80.0
Severe disease (Difficulty in Breathing)	1	10.0
Any household members tested positive for COVID-19		
No	403	95.0
Yes	21	5.0
Any household deaths due to COVID-19 (Only people they lived with: Excluding non-resident friends, relatives and neighbours)		
No	419	98.8
Yes	5	1.2
Any household members vaccinated against COVID-19		
Don’t know	78	18.4
No	126	29.7
Yes	220	51.9
Relationship of vaccinated household members to respondent††,, *n* = 220		
Husband/wife/spouse/partner	205	93.2
Children	8	3.6
Sister/brother	23	10.5
Parents	27	12.3
Other relatives including aunties, uncles, in-laws	4	1.8
Grandparents	1	0.5
Live-in house workers	2	0.9
Grand children	0	0.0

† Gravidity, duration of current pregnancy, and twin/multiple pregnancy were assessed among pregnant women only (*n* = 212). ‡ Duration of current lactation was assessed among lactating women only (*n* = 212). § Type of chronic illness was reported among participants with chronic illness (*n* = 34). ¶ Year of chronic illness diagnosis was available for 27 of the 34 participants with chronic illness. ** History of hospitalization and disease severity were assessed among participants with confirmed COVID-19 infection (*n* = 10). †† Multiple responses were allowed for the relationship of vaccinated household members to the respondent; therefore, percentages may exceed 100%

### Level of uptake and COVID-19 vaccination acceptability among study participants

Among 424 study participants, 219 (51.6%) had received at least one dose of COVID-19 vaccine; of these, 77 (35.2%) had received only one dose, 122 (55.7%) two doses, and 20 (9.1%) had received boosters. Many of the vaccinated participants, 99/219 (45.2%) were not aware of the type of COVID-19 vaccine they had received while 54 (24.7%) had received AstraZeneca COVID-19 vaccine.

Most participants, 148/219 (67.6%) received their first dose of COVID-19 vaccine in 2021. Only 4/212(1.9%) had been vaccinated for COVID-19 while lactating and, none received COVID-19 vaccines when pregnant.

Among the 205 unvaccinated, 67 (32.7%) were willing to receive the COVID-19 vaccine. Only 43/77 (55.8%) of those who received one dose and 75/122 (61.5%) of those who received two doses expressed willingness to receive additional COVID-19 vaccines doses. All those who got boosters were willing to get additional doses. The most common reasons for unwillingness to receive COVID-19 vaccines included fear of side effects and safety concerns during pregnancy and lactation. Overall, the willingness to receive the COVID-19 vaccine among all participants, regardless of vaccination status, was 205 out of 424 (48.4%).

In the past three years, 399 (94.1%) participants reported having received Tetanus Toxoid vaccines. Nearly half of the participants 207 (48.8%) were not willing to have any other vaccines while pregnant or lactating. Other vaccination related factors are summarized in Table 3.

**Table 3:** Level of uptake and vaccination acceptability among study participants

Characteristic, *n* = 424	Frequency	Percentage (95% CI)				
COVID-19 vaccination status (at least one dose)						
Yes	219	51.6 (43.6–53.1)				
No	205	48.4 (46.9–56.4)				
	Frequency (Percentage)					
COVID-19 vaccine doses received, *n* = 219						
Booster dose	20	9.1				
One dose	77	35.2				
Two doses	122	55.7				
Type of COVID-19 vaccine received‡, *n* = 219	AstraZeneca 54 (24.7)	Johnson 33 (15.1)	Moderna 6 (2.7)	Pfizer 31 (14.2)	Don’t know 99 (45.2)	Sinovac 1 (0.5)
Dose 1 only	17 (31.5)	27 (81.8)	-	8 (25.8)	29 (29.3)	-
Dose 2 only	5 (9.3)	-	-	2 (6.5)	11 (11.1)	-
Booster only	-	1 (3.0)	-	-	-	-
Dose1 & dose2	29 (53.7)	3 (9.1)	5 (83.3)	17 (54.8)	46 (46.5)	1
Dose1 &dose2 &booster	3 (5.6)	2 (6.1)	1 (16.7)	4 (12.9)	9 (9.1)	-
	Vaccination year					
Vaccination dose	2021	2022	2023			
First dose, *n* = 219	148 (67.6)	67 (30.6)	4 (1.8)			
Second dose, *n* = 142	88 (62.0)	52 (36.6)	2 (1.4)			
Booster dose, *n* = 20	12 (60.0)	8 (40.0)	-			
Willingness to receive COVID-19 vaccine						
If received COVID-19 one dose, willingness to receive the second dose, *n* = 77						
Not sure	16	20.8				
Not willing	18	23.4				
Willing	43	55.8				
If not willing, reasons why, *n* = 18						
COVID-19 ended	1	5.6				
Got side effects/Fear of side effects	6	33.3				
Due to my condition (pregnant/lactating)	3	16.7				
Believes one dose is enough	2	11.1				
Got single dose vaccine (Johnson&Johnson), does not think they need another dose	6	33.3				
If received two COVID-19 doses, willingness to receive additional shots (boosters), *n* = 122						
Not sure	19	15.6				
Not willing	28	22.9				
Willing	75	61.5				
If not sure/not willing, reasons why, *n* = 28						
COVID-19 ended	1	3.6				
Fear of side effects	4	14.3				
Due to my condition, still (pregnant/lactating)	5	17.9				
Two doses are enough, I completed	11	39.3				
Tired	2	7.1				
Don’t know where to get it from	3	10.7				
I never got COVID-19	2	7.1				
If received booster doses, willingness to receive additional shots, *n* = 20						
Not sure	0	0				
Not willing	0	0				
Willing	20	100				
If has never received any doses, currently willing to receive the COVID-19 vaccine, *n* = 205						
Not sure	49	23.9				
Not willing	89	43.4				
Willing	67	32.7				
When they are willing to get the COVID-19 vaccine, *n* = 67						
After birth	5	7.5				
After lactation	19	28.4				
Anytime including now	20	29.8				
Not sure	22	32.8				
If fully given information	1	1.5				
How many Weeks after birth they are willing to get the COVID-19 vaccine, *n* = 5						
2	4	80.0				
3	1	20.0				
How many Weeks after lactation they are willing to get the COVID-19 vaccine, *n* = 19						
< 12	9	47.4				
12–20	4	21.1				
> 20	6	31.6				
If no, why are you not willing to get the COVID-19 vaccine, *n* = 89						
Perceived that the COVID-19 pandemic had ended	15	16.9				
Fear of side effects	19	21.3				
Fear of injections	12	13.5				
I never got COVID-19	5	5.6				
Concerns about vaccine safety during pregnancy/lactation	21	23.6				
Too many myths about the vaccines	13	14.6				
Don’t have a national ID	4	4.5				
Overall willingness to currently receive a COVID-19 vaccine regardless of COVID-19 vaccination status						
	Frequency	Percentage (95% CI)				
Not sure/Not willing	219	51.6 (46.8–56.4)				
Willing	205	48.4 (43.6–53.1)				
In 2020/2021, when we had many cases and deaths due to COVID-19 and implemented lockdowns and restrictions, were you willing to receive the vaccine then, *n* = 205 (never vaccinated with any dose COVID-19)						
	Frequency	Percentage				
Not sure	38	18.5				
Not willing	102	49.8				
Willing	65	31.7				
Other vaccines						
What other vaccines have you received since January 2020?†						
None	19	4.5				
Tetanus toxoid	399	94.1				
Rabies	3	0.7				
Typhoid	6	1.4				
Yellow fever	11	2.6				
Hepatitis B	27	6.4				
Regardless of COVID-19 vaccine willingness, what vaccines are they willing to receive currently (Now)†						
None	207	48.8				
Tetanus toxoid	112	26.4				
Rabies	13	3.1				
Typhoid	25	5.9				
Yellow fever	33	7.8				
Meningitis	10	2.4				
Hepatitis B	80	18.9				

† Multiple responses were allowed; percentages may exceed 100%. ‡ Some participants received more than one COVID-19 vaccine type across doses; therefore, frequencies for vaccine type may exceed the total number of vaccinated participants (*n* = 219)

### Contextual, individual, group and vaccine-specific issues

Among 424 respondents, 99 (23.3%) had ever received some information from the health workers on COVID-19 vaccination among pregnant/lactating women, and 132 (31.1%) had ever attended health education programs or teachings on COVID-19 vaccination at the health facility. Most of the participants 360 (84.9%) reported ownership or regularly access to television, radio mobile phone (smart), internet or newspapers. Overall, 201 (47.4%) did not use social media platforms while 211 (49.8%) used WhatsApp, 164 (38.9%) used Facebook, 88 (20.8%) used Twitter, 55 (13.0%) used Tik Tok, 41 (9.7%) used YouTube and 24 (5.7%) used Snapchat. Among 223 participants who used social media, 149 (66.8%) obtained information related to COVID-19 vaccination from social media, and only 66 (29.6%) reported that they shared information related to COVID-19 vaccination on social media.

When asked about who they trust the most for information on COVID-19 vaccination, 380 (89.6%) trusted health workers including community health workers, 34 (8.0%) family, friends, relatives, and neighbours, 16 (3.8%) media such as radio, TV, and internet, 21 (4.9%) religious leaders and 6 (1.4%) leaders including politicians and local leaders. Only 108 (25.5%) participants reported that they believed some media reports of deaths following COVID-19 vaccination. Only 22 (5.2%) knew someone who hadn’t taken the COVID-19 vaccine due to religious reasons and only 11 (2.6%) knew someone who hadn’t taken the COVID-19 vaccine due to cultural/tribal/ethnicity reasons. Most participants, 400 (94.3%) reported that their religion recommended vaccination, while 312 (73.6%) reported that their culture/tribe/ethnicity recommended vaccination. Only 53 (12.5%) reported that they would refuse the COVID-19 vaccine if the vaccinator was from another religion and only 25 (5.9%) would refuse if the vaccinator was from another culture/tribe/ethnicity.

Only 113 (26.6%) participants trusted that pharmaceutical companies are providing safe and effective COVID-19 vaccines for pregnant and lactating women. Furthermore, 146 (34.4%) believed there are other ways COVID-19 would be prevented instead of the vaccine. Only 44 (10.4%) felt that they had enough information about COVID-19 vaccines and their safety in pregnant or lactating women, while 252 (59.4%) were very concerned that some of the COVID-19 vaccines may not be safe for pregnant or lactating women and only 122 (28.7%) thought that the COVID-19 vaccination would still be needed after the COVID-19 epidemic ends. Only 96 (22.6%) believed that vaccines in general were for them and their unborn/breastfeeding babies and 59 (13.9%) believed the COVID-19 vaccine was safe for them and their unborn/breastfeeding baby (supplementary material 1).

When asked about which mode of vaccination (injection or oral) they preferred for any vaccine, 227 (53.5%) preferred injections or oral, 142 (33.5%) injections only and 55 (13.0%) oral only. About where they preferred to receive the COVID-19 vaccine, 235 (55.4%) preferred a health centre, 122 (28.8%) from schools or institution-based programs, 68 (16.0%) mass campaign in community, 60 (14.2%) door to door and 26 (6.1%) personal doctor. Only 92 (21.7%) reported that they would follow the recommended schedule for COVID-19 vaccination (8 weeks after the first dose) while if the COVID-19 vaccine was offered at a cost, only 50 (11.8%) would still get it. Other participant responses are presented in Supplementary material 1.

### Factors associated with COVID-19 vaccine uptake and willingness

Bivariate and multivariable modified Poisson regression analyses were conducted to identify factors associated with COVID-19 vaccine uptake and willingness to receive COVID-19 vaccines. Results are presented below.

### Factors associated with uptake of COVID-19 vaccines among study participants

In the bivariate analysis, COVID-19 vaccine uptake was associated with having heard about health insurance, ever testing for COVID-19, willingness to receive an Ebola vaccine, having household members vaccinated against COVID-19, receiving information from health workers about COVID-19 vaccination, receiving information from health workers about COVID-19 vaccination among pregnant and lactating women, attending health education programs on COVID-19 vaccination at the health facility, and having ever experienced a bad reaction after immunization.

In the multivariable analysis, after adjusting for covariates, COVID-19 vaccine uptake remained significantly associated with ever testing for COVID-19, regardless of test result (aPR = 1.92, 95% CI: 1.45–2.54), and having a household member vaccinated against COVID-19 (aPR = 1.38, 95% CI: 1.03–1.84). Other variables that were significant in the bivariate analysis were no longer independently associated with COVID-19 vaccine uptake after adjustment (Table 4).

**Table 4:** Factors associated with uptake of COVID-19 vaccine (at least one dose) among study participants

Variable, (*n* = 424)	Not vaccinated, *n* = 205 (%)	Vaccinated, *n* = 219 (%)	Crude PR (95% CI), *P* value	Adjusted PR (95% CI), *P* value
Pregnant/lactating				
Pregnant	99 (46.7)	113 (53.3)	1.00 (ref)	
Lactating	106 (50.0)	106 (50.0)	0.94 (0.72–1.22), 0.636	
Age categories (years)				
< 18	6 (85.7)	1 (14.3)	0.25 (0.04–1.86), 0.178	
18–25	91 (49.2)	94 (50.8)	0.91 (0.66–1.28), 0.599	
26–30	64 (48.1)	69 (51.9)	0.93 (0.66–1.33), 0.705	
31–49	44 (44.4)	55 (55.6)	1.00 (ref)	
District of residence				
Kampala	82 (45.8)	97 (54.2)	1.00 (ref)	
Wakiso	108 (49.1)	112 (50.9)	0.94 (0.72–1.23), 0.653	
Mukono	4 (57.1)	3 (42.9)	0.79 (0.25–2.49), 0.689	
Others	11 (61.1)	7 (38.9)	0.72 (0.33–1.54), 0.397	
Distance from home to the nearest public health facility that offers COVID-19 vaccination				
≤ 5 km	91 (49.2)	94 (50.8)	0.97 (0.74–1.26), 0.832	
> 5 km	114 (47.7)	125 (52.3)	1.00 (ref)	
Tribe				
Baganda	125 (50.8)	121 (49.2)	1.00 (ref)	
Banyankore	32 (48.5)	34 (51.5)	1.05 (0.72–1.53), 0.812	
Basoga	12 (48.0)	13 (52.0)	1.06 (0.59–1.87), 0.849	
Others	36 (41.4)	51 (58.6)	1.19 (0.86–1.65), 0.293	
Marital status				
Married/cohabiting	185 (48.3)	198 (51.7)	1.00 (ref)	
Separated/widowed/divorced	4 (57.1)	3 (42.9)	0.83 (0.26–2.59), 0.747	
Single/never married	16 (47.1)	18 (52.9)	1.02 (0.63–1.66), 0.923	
Highest Education level achieved				
None/Primary	62 (53.9)	53 (46.1)	1.00 (ref)	
Secondary	117 (47.6)	129 (52.4)	1.14 (0.83–1.56), 0.429	
Tertiary/vocational/University	26 (41.3)	37 (58.7)	1.27 (0.84–1.94), 0.258	
Religion				
Adventist	6 (60.0)	4 (40.0)	0.68 (0.25–1.84), 0.445	
Anglican	30 (44.1)	38 (55.9)	0.95 (0.65–1.38), 0.773	
born-again	53 (55.8)	42 (44.2)	0.75 (0.52–1.08), 0.120	
Catholic	63 (40.9)	91 (59.1)	1.00(ref)	
Muslim	52 (54.2)	44 (45.8)	0.76 (0.54–1.11), 0.116	
Faith of Unity	1 (100)	-	-	
House hold head				
No	181 (47.3)	202 (52.7)	1.00 (ref)	
Yes	24 (58.5)	17 (41.5)	0.78 (0.48–0.61), 0.341	
Number of biological children (Alive only)				
0	29 (45.3)	35 (54.7)	1.00 (ref)	
1–5	169 (49.1)	175 (50.9)	0.93 (0.65–1.34), 0.696	
≥ 6	7 (43.7)	9 (56.3)	1.03 (0.49–2.14), 0.940	
House hold size (number of live in occupants)				
1–5	173 (48.7)	182 (51.3)	1.00 (ref)	
≥ 6	32 (46.4)	37 (53.6)	1.05 (0.73–1.48), 0.803	
Employment status of respondent (Has a job or business or works somewhere)				
No	142 (50.4)	140 (49.6)	1.00 (ref)	
Yes	63 (44.4)	79 (55.6)	1.12 (0.85–1.47), 0.418	
Heard about health insurance				
No	169 (52.3)	154 (47.7)	1.00 (ref)	
Yes	36 (35.6)	65 (64.4)	1.35 (1.01–1.80), 0.043	
Had any health insurance since 2020				
No	203 (48.3)	217 (51.7)	1.00 (ref)	
Yes	2 (50.0)	2 (50.0)	0.96 (0.24–0.59), 0.963	
Parity				
0	27 (48.2)	29 (51.8)	1.00 (ref)	
1–3	150 (50.3)	148 (49.7)	0.96 (0.64–1.43), 0.837	
4–5	22 (41.5)	31 (58.5)	1.13 (0.68–1.87), 0.637	
≥ 6	6 (35.3)	11 (64.7)	1.25 (0.62–2.50), 0.529	
How many pregnancies between January 2020 and now? including miscarriages				
1	160 (47.1)	180 (52.9)	1.00 (ref)	
2	35 (53.0)	31 (47.0)	0.88 (0.61–1.29), 0.538	
≥ 3	10 (55.6)	8 (44.4)	0.84 (0.41–1.70), 0.628	
Chronic illness(es)				
Yes	17 (50.0)	17 (50.0)	1.04 (0.63–1.70), 0.889	
No	188 (48.2)	202 (51.8)	1.00 (ref)	
Ever tested for COVID-19				
No	181 (61.6)	113 (38.4)	1.00 (ref)	1.00 (ref)
Yes	24 (18.5)	106 (81.5)	2.12 (1.63–2.77), 0.000	1.92 (1.45–2.54), 0.000
History of a confirmed Positive COVID-19 test				
No	203 (49.0)	211 (51.0)	1.00 (ref)	
Yes	2 (20.0)	8 (80.0)	1.57 (0.77–3.18), 0.211	
Any household members tested positive for COVID-19				
No	200 (49.6)	203 (50.4)	1.00 (ref)	
Yes	5 (23.8)	16 (76.2)	1.51 (0.91–2.52), 0.111	
Any household deaths due to COVID-19 (Only people they lived with: Excluding non-resident friends, relatives and neighbours)				
No	204 (48.7)	215 (51.3)	1.00 (ref)	
Yes	1 (20.0)	4 (80.0)	1.56 (0.58–4.19), 0.379	
Received some other vaccines since January 2020				
No other vaccines	201 (49.6)	204 (50.4)	1.00 (ref)	
Yes	4 (21.1)	15 (78.9)	1.57 (0.93–2.65), 0.093	
If there was an Ebola Virus Disease vaccine, would you currently be willing to receive it?				
Not sure/Not willing	103 (59.9)	69 (40.1)	1.00(ref)	
Willing	102 (40.5)	150 (59.5)	1.48 (1.12–1.97), 0.007	
Have any household members been vaccinated against COVID-19?				
Don’t know/No	126 (61.8)	78 (38.2)	1.00 (ref)	1.00 (ref)
Yes	79 (35.9)	141 (64.1)	1.67 (1.27–2.21), 0.000	1.38 (1.03–1.84), 0.030
While at the health facility, did you EVER receive any information from the health workers on COVID-19 vaccination?				
No	152 (60.8)	98 (39.2)	1.00 (ref)	
Yes	53 (30.5)	121 (69.5)	1.77 (1.36–2.32), 0.000	
Did you EVER receive any information from the health workers on COVID-19 vaccination among pregnant/lactating women?				
No	173 (53.2)	152 (46.8)	1.00 (ref)	
Yes	32 (32.3)	67 (67.7)	1.45 (1.08–1.93), 0.012	
Did you EVER attend any health education programs or teachings on COVID-19 vaccination at the health facility?				
No	159 (54.5)	133 (45.5)	1.00 (ref)	
Yes	46 (34.8)	86 (65.2)	1.43 (1.09–1.87), 0.010	
Does participant own or regularly has access to any of these? Television Radio Mobile phone (smart) Internet Newspapers				
No	41 (64.1)	23 (35.9)	1.00 (ref)	
Yes	164 (45.6)	196 (54.4)	1.55 (0.98–2.33), 0.059	
Do you obtain information related to COVID-19 vaccination on social media?				
No	138 (50.2)	137 (49.8)	1.00 (ref)	
Yes	67 (45.0)	82(55.0)	1.10 (0.84–1.45), 0.476	
Have you EVER experienced any bad reaction after any immunization?				
No/Unsure	196 (50.6)	191 (49.4)	1.00 (ref)	
Yes	9(24.3)	28 (75.7)	1.53 (1.03–2.28), 0.035	
Which mode of vaccination (injection or oral) do you PREFER? (For any vaccine)				
Injections or oral	118 (52.0)	109 (48.0)	1.00 (ref)	
Injections only	58 (40.9)	84 (59.1)	1.23 (0.93–1.63), 0.151	
Oral only	29 (52.7)	26 (47.3)	0.98 (0.64–1.51), 0.943	

*PR* Prevalence Ratio

### Factors associated with willingness to receive COVID-19 vaccines among study participants

In the bivariate analysis, willingness to receive COVID-19 vaccines was associated with being a household head, willingness to receive an Ebola vaccine, having household members vaccinated against COVID-19, previous COVID-19 vaccine uptake, receiving information from health workers about COVID-19 vaccination, and attending health education programs on COVID-19 vaccination at the health facility.

In the multivariable analysis, after adjusting for covariates, willingness to receive COVID-19 vaccines remained significantly associated with being a household head and previous COVID-19 vaccine uptake. Participants who were household heads were less likely to be willing to receive COVID-19 vaccines compared with those who were not household heads (aPR = 0.46, 95% CI: 0.24–0.89). Participants who had previously received at least one dose of COVID-19 vaccine were more likely to be willing to receive additional COVID-19 vaccine doses compared with those who were unvaccinated (aPR = 1.88, 95% CI: 1.41–2.52). Other variables that were significant in the bivariate analysis were no longer independently associated with willingness to receive COVID-19 vaccines after adjustment (Table 5).

**Table 5:** Factors associated with willingness to receive the COVID-19 vaccine among study participants

Variable, (*n* = 424)	Not willing, *n* = 219 (%)	Willing to receive, *n* = 205 (%)	Crude PR (95% CI), *P* value	Adjusted PR (95% CI), *P* value
Pregnant/lactating				
Pregnant	112 (52.8)	100 (47.2)	1.00 (ref)	
Lactating	107 (50.5)	105 (49.5)	1.05 (0.79–1.38), 0.727	
Socio demographic factors				
Age categories (years)				
< 18	1 (14.3)	6 (85.7)	1.00 (ref)	
18–25	106 (57.3)	79 (42.7)	0.49 (0.22–1.14), 0.100	
26–30	66 (49.6)	67 (50.4)	0.58 (0.25–1.35), 0.212	
31–49	46 (46.5)	53 (53.5)	0.62 (0.27–1.45), 0.275	
District of residence				
Kampala	97 (54.2)	82 (45.8)	1.00 (ref)	
Wakiso	111 (50.5)	109 (49.5)	1.08 (0.81–1.44), 0.592	
Mukono	1 (14.3)	6 (85.7)	1.87 (0.82–4.28), 0.138	
Others	10 (55.6)	8 (44.4)	0.97 (0.46-2.00), 0.935	
Distance from home to the nearest public health facility that offers COVID-19 vaccination				
≤ 5 km	98 (53.0)	87 (47.0)	1.00 (ref)	
> 5 km	121 (50.6)	118 (49.4)	0.95 (0.72–1.25), 0.731	
Tribe				
Baganda	125 (50.8)	121 (49.2)	1.00 (ref)	
Banyankore	34 (51.5)	32 (48.5)	0.98 (0.67–1.45), 0.942	
Basoga	12 (48.0)	13 (52.0)	1.06 (0.59–1.87), 0.849	
Others	48 (55.2)	39 (44.8)	0.91 (0.64–1.31), 0.614	
Marital status				
Married/cohabiting	196 (51.2)	187 (48.8)	1.00 (ref)	
Separated/widowed/divorced	6 (85.7)	1 (14.3)	0.29 (0.04–2.09), 0.220	
Single/never married	17 (50.0)	17 (50.0)	1.02 (0.62–1.68), 0.925	
Highest Education level achieved				
None/Primary	60 (52.2)	55 (47.8)	1.00 (ref)	
Secondary	126 (51.2)	120 (48.8)	1.02 (0.74–1.40), 0.903	
Tertiary/vocational/University	33 (52.4)	30 (47.6)	0.99 (0.64–1.55), 0.985	
Religion				
Adventist	8 (80.0)	2 (20.0)	0.40 (0.09–1.65), 0.207	
Anglican	32 (47.1)	36 (52.9)	1.07 (0.72–1.59), 0.729	
born-again	52 (54.7)	43 (45.3)	0.92 (0.63–1.33), 0.651	
Catholic	78 (50.6)	76 (49.4)	1.00 (ref)	
Muslim	48 (50.0)	48 (50.0)	1.01 (0.71–1.45), 0.943	
Faith of Unity	1 (100)	0	-	
House hold head				
No	187 (48.8)	196 (51.2)	1.00 (ref)	1.00 (ref)
Yes	32 (78.1)	9 (21.9)	0.43 (0.22–0.84), 0.013	0.46 (0.24–0.89), 0.023
Number of biological children (Alive only)				
0	38 (59.4)	26 (40.6)	1.00 (ref)	
1–5	172 (50.0)	172 (50.0)	1.23 (0.81–1.86), 0.324	
≥ 6	9 (56.3)	7 (43.7)	1.07 (0.46–2.48), 0.862	
House hold size (number of live in occupants)				
1–5	188 (53.0)	167 (47.0)	1.00 (ref)	
≥ 6	31 (44.9)	38 (55.1)	1.17 (0.82–1.66), 0.381	
Employment status of respondent (Has a job or business or works somewhere)				
No	139 (49.3)	143 (50.7)	1.00 (ref)	
Yes	80 (56.3)	62 (43.7)	0.86 (0.64–1.16), 0.325	
Heard about health insurance				
No	172 (53.3)	151 (46.7)	1.00 (ref)	
Yes	47 (46.5)	54 (53.5)	1.14 (0.83–1.56), 0.397	
Had any health insurance since 2020				
No	216 (51.4)	204 (48.6)	1.00 (ref)	
Yes	3 (75.0)	1 (25.0)	0.51 (0.07–3.67), 0.508	
Parity				
0	33 (58.9)	23 (41.1)	1.00 (ref)	
1–3	155 (52.0)	143 (48.0)	1.17 (0.75–1.81), 0.489	
4–5	21 (39.6)	32 (60.4)	1.47 (0.86–2.51), 0.159	
≥ 6	10 (58.8)	7 (41.2)	1.00 (0.43–2.34), 0.995	
How many pregnancies between January 2020 and now? including miscarriages				
1	170 (50.0)	170 (50.0)	1.00 (ref)	
2	37 (56.1)	29 (43.9)	0.88 (0.59–1.30), 0.520	
≥ 3	12 (66.7)	6 (33.3)	0.67 (0.29–1.50), 0.329	
Chronic illness(es)				
Yes	23 (67.6)	11 (32.4)	1.00 (ref)	
No	196 (50.3)	194 (49.7)	1.54 (0.84–2.82), 0.165	
Ever tested for COVID-19				
No	161 (54.8)	133 (45.2)	1.00 (ref)	
Yes	58 (44.6)	72 (55.4)	1.22 (0.92–1.63), 0.167	
History of a confirmed Positive COVID-19 test				
No	216 (52.2)	198 (47.8)	1.00 (ref)	
Yes	3 (30.0)	7 (70.0)	1.46 (0.69–3.11), 0.322	
Any household members tested positive for COVID-19				
No	204 (50.6)	199 (49.4)	1.00 (ref)	
Yes	15 (71.4)	6 (28.6)	0.58 (0.26–1.30), 0.187	
Any household deaths due to COVID-19 (Only people they lived with: Excluding non-resident friends, relatives and neighbours)				
No	216 (51.5)	203 (48.5)	1.00 (ref)	
Yes	3 (60.0)	2 (40.0)	0.83 (0.21–3.32), 0.787	
Received some other vaccines since January 2020				
No other vaccines	212 (52.4)	193 (47.6)	1.00 (ref)	
Yes	7 (36.8)	12 (63.2)	1.32 (0.74–2.37), 0.344	
If there was an Ebola Virus Disease vaccine, would you currently be willing to receive it?				
Not sure/Not willing	135 (78.5)	37 (21.5)	1.00 (ref)	
Willing	84 (33.3)	168 (66.7)	3.10 (2.17–4.42), 0.000	
Have any household members been vaccinated against COVID-19?				
Don’t know/No	122 (59.8)	82 (40.2)	1.00 (ref)	
Yes	97 (44.1)	123(55.9)	1.39 (1.05–1.84), 0.021	
Previous COVID-19 vaccinated (uptake)				
No	138 (67.3)	67 (32.7)	1.00 (ref)	1.00 (ref)
Yes	81 (37.0)	138 (63.0)	1.93 (1.44–2.58), 0.000	1.88 (1.41–2.52), 0.000
While at the health facility, did you EVER receive any information from the health workers on COVID-19 vaccination?				
No	145 (58.0)	105 (42.0)	1.00 (ref)	
Yes	74 (42.5)	100 (57.5)	1.37 (1.04–1.79), 0.025	
Did you EVER receive any information from the health workers on COVID-19 vaccination among pregnant/lactating women?				
No	175 (53.8)	150 (46.2)	1.00 (ref)	
Yes	44 (44.4)	55 (55.6)	1.20 (0.88–1.64), 0.240	
Did you EVER attend any health education programs or teachings on COVID-19 vaccination at the health facility?				
No	167 (57.2)	125 (42.8)	1.00 (ref)	
Yes	52 (39.4)	80 (60.6)	1.42 (1.07–1.87), 0.015	
Does participant own or regularly has access to any of these? Television Radio Mobile phone (smart) Internet Newspapers				
No	41 (64.1)	23 (35.9)	1.00 (ref)	
Yes	178 (49.4)	182 (50.6)	1.41 (0.91–2.17), 0.123	
Do you obtain information related to COVID-19 vaccination on social media?				
No	143 (52.0)	132 (48.0)	1.00 (ref)	
Yes	76 (51.0)	73 (49.0)	1.02 (0.77–1.36), 0.888	
Have you EVER experienced any bad reaction after any immunization?				
No/Unsure	205 (53.0)	182 (47.0)	1.00 (ref)	
Yes	14 (37.8)	23 (62.2)	1.32 (0.86–2.04), 0.207	
Did you get a bad reaction after the first COVID-19 dose?				
No/Unsure	210 (52.1)	193 (47.9)	1.00 (ref)	
Yes	9 (42.9)	12 (57.1)	1.19 (0.66–2.14), 0.553	
Which mode of vaccination (injection or oral) do you PREFER? (For any vaccine)				
Injections or oral	129 (56.8)	98 (43.2)	1.00 (ref)	
Injections only	60 (42.3)	82 (57.7)	1.34 (0.99–1.79), 0.052	
Oral only	30 (54.5)	25 (45.5)	1.05 (0.68–1.63), 0.818	

*PR* Prevalence Ratio

## Discussion

Our study provides valuable insights into the factors influencing COVID-19 vaccine uptake and acceptability among pregnant and lactating women attending a tertiary public hospital in Kampala, Uganda. Despite the established benefits of COVID-19 vaccination during pregnancy and lactation, our findings indicate suboptimal coverage of COVID-19 vaccines among study participants. Our findings are in agreement with a systematic review of studies conducted between March 2020 and April 2023 which reported that COVID-19 vaccine uptake during pregnancy was low in Sub-Saharan Africa at (14.4–28)% [12].

None of the women in our study received a COVID-19 vaccine during pregnancy, whilst very few received it during lactation. This can be explained by the fact that there were no educational campaigns and communication strategies for COVID-19 vaccination tailored to pregnant and lactating women as part of Uganda’s COVID-19 vaccination program [13]. However, other factors likely contributed. During the early phases of vaccine rollout, pregnant and lactating women were excluded from most COVID-19 vaccine clinical trials, resulting in limited initial safety data for these populations and uncertainty among both women and healthcare providers [14, 15]. In addition, misinformation and rumours circulating during the pandemic, including concerns that COVID-19 vaccines could cause infertility, miscarriage, or harm to the unborn child, may have further contributed to vaccine hesitancy among pregnant women in Uganda and other African settings [16, 17].

Our study found that only 32.7% of unvaccinated participants were willing to receive the COVID-19 vaccine. Acceptability was also suboptimal among those who had received one (55.8%) or two (61.5%) doses, but 100% of participants who received booster doses were willing to receive additional doses. This suggests that women who have taken up the vaccine could be involved as ‘expert patients’ in campaigns to help encourage their peers that the vaccination is helpful and safe, potentially enhancing vaccine acceptability and uptake among pregnant and lactating women.

Our findings show that COVID-19 vaccine uptake was associated with history of testing for COVID-19 and having a vaccinated household member. This highlights the influence of social networks and household dynamics on vaccine decision-making. Pregnant and lactating women with a history of COVID-19 testing or with vaccinated household members were more likely to accept COVID-19 vaccines, suggesting the importance of peer influence and social norms in shaping vaccine behaviors. Similar findings have been reported in studies from Sub-Saharan Africa showing that family members, peers, and household decision-makers play an important role in shaping perceptions of COVID-19 vaccination and vaccine uptake [18].

In Uganda, COVID-19 testing was challenging to access. A Polymerase Chain Reaction (PCR) test cost around 250,000 Ugandan shillings (approximately 68 USD), and most people had to pay for their tests, except in a few public health facilities that often-faced stockouts [19]. Limited testing supplies, mostly imported, and high costs discouraged many Ugandans from getting tested. Free testing was available only to specific groups, such as health workers and individuals seeking care at public health facilities [19, 20]. Consequently, even those with severe symptoms often faced barriers to testing. This context emphasizes the significant influence of testing accessibility on vaccine uptake behaviors.

Similar to our study findings, a study among health workers in Nigeria showed that those who had been tested for COVID-19 were more likely (aOR:7.64; *p* < 0.001) to accept the vaccine [21].

Interestingly, our study identified a significant association between being a household head and willingness to receive COVID-19 vaccines, further indicating the potential role of household dynamics in shaping vaccine attitudes. This finding may suggest that women who are household heads do not face the same barriers related to seeking permission from a husband or male partner. It may also reflect altered priorities due to being the sole parent, and understanding this area could be a future research priority. Our findings are in line with findings from a survey involving six countries in East and West Africa which reported that social ties and perceptions as well as intra-household power relations matter for COVID-19 vaccine take-up [22]. Future research could delve deeper into how intra-household decision-making processes affect vaccine acceptance among pregnant/lactating women.

Our findings show that 94.1% of the women had received TT vaccines in the past three years. TT vaccines have been administered to pregnant women for many years with minimal safety and community concerns, making them more trusted than newer vaccines like COVID-19 vaccines. There was a low trust in the safety of vaccines in general and COVID-19 vaccines for women and their unborn /breastfeeding babies. However, nearly half of the women (48.8%) were not willing to receive any other vaccines while pregnant or lactating, possibly due to concerns about the safety of new vaccines, lack of information, or cultural beliefs. Understanding these reasons is crucial for developing strategies to improve vaccine acceptance in this population.

Concerns about vaccine safety during pregnancy and breastfeeding have been consistently reported across Africa and globally as major drivers of COVID-19 vaccine hesitancy. Studies conducted among pregnant women in Ethiopia, Nigeria, and Ghana found that fears of adverse effects on the mother, fetus, or breastfeeding infant were among the most frequently cited reasons for declining vaccination [23–26]. Similar findings have been reported in systematic reviews showing that uncertainty regarding vaccine safety, particularly during the early stages of vaccine rollout when pregnant women were excluded from many clinical trials, contributed substantially to low vaccine acceptance [27–29]. Our findings therefore align with a broader body of evidence indicating that safety concerns remain a key barrier to maternal immunization and should be addressed through targeted risk communication and counselling during antenatal and postnatal care.

In addition, we established that while health workers were most trusted source of information on COVID-19, the reach of their education campaigns on COVID-19 including specific information for pregnant and breastfeeding women were poor. Only 16.5% of the study participants felt that COVID-19 vaccine side effects had been discussed openly by the public health authorities and 18.4% had received information on adverse events. Future health promotions should monitor the reach of health worker-led education campaigns.

Our finding that health workers were the most trusted source of information is consistent with studies conducted among pregnant women in several African settings. Studies from Ethiopia, Nigeria, and Cameroon have reported that recommendations from healthcare providers were among the strongest predictors of COVID-19 vaccine acceptance during pregnancy, while lack of counselling from health professionals contributed to hesitancy [30–32]. In many settings, women who received vaccine information directly from healthcare workers were significantly more likely to express willingness to be vaccinated compared with those relying primarily on social media or informal community sources [32, 33]. These findings reinforce the importance of equipping maternal health providers with up-to-date vaccine information and communication skills to address concerns regarding vaccine safety and effectiveness.

COVID-19 health literacy campaigns in Uganda engaged a lot of political, religious leaders, however women reported that trust in these figures is low; future campaigns should strategically engage more health workers. There was also low trust in information from media outlets. While nearly half of the women did not use social media, the majority who had access had obtained information on COVID-19 from there. This is in line with the heightened role of social media in health literacy that COVID-19 exposed [34–36].

The low willingness to pay for the COVID-19 vaccine if it was offered at a cost could have implications for future strategies for public roll-out of vaccines. Previous studies have shown that willingness to pay for COVID-19 vaccines varies across settings and is generally higher among individuals with greater income levels and higher perceived risk of infection [37, 38]. In our setting where the employment rate was low with a median monthly income of 50 USD among the study participants, and where the perceived risk of COVID-19 was low, the low reported rate of willingness to pay for the vaccine may be easier to understand .

Interestingly, willingness to receive a hypothetical Ebola vaccine was strongly associated with both COVID-19 vaccine uptake and willingness to receive COVID-19 vaccines at the bivariate level. This finding likely reflects underlying confidence in vaccination rather than attitudes toward a specific vaccine. Uganda has experienced multiple Ebola outbreaks, including the 2022 Sudan Ebola Virus Disease outbreak, which may have heightened awareness of the severity of Ebola and the potential value of vaccination. Consistent with this observation, our recent analysis of the same study population found that 59.4% of pregnant and lactating women were willing to receive an Ebola vaccine, and that previous COVID-19 vaccination was independently associated with willingness to receive an Ebola vaccine (aPR = 1.32; 95% CI: 1.02–1.70) [39]. These findings suggest that vaccine confidence and positive attitudes toward immunization may extend across diseases rather than being vaccine-specific. Individuals who are generally confident in vaccines and public health interventions may therefore be more willing to accept both COVID-19 and Ebola vaccines.

### Limitations

The limitations of our study include its cross-sectional design, which prohibits causal inference, and the reliance on self-reported data, which may be subject to recall bias and social desirability bias. Additionally, our study was conducted at a single tertiary public hospital in Kampala, limiting the generalizability of our findings to other settings in Uganda. Furthermore, the study was conducted between May and July 2023, when COVID-19 was no longer considered a global public health emergency and public health messaging regarding COVID-19 vaccination had declined. This context may have influenced participants’ perceptions of COVID-19 risk and their willingness to receive COVID-19 vaccines. Because some questions required participants to recall vaccination experiences and attitudes from earlier phases of the pandemic, recall bias may have affected the accuracy of some responses.

## Conclusion

COVID-19 vaccine acceptance among pregnant and lactating women remains suboptimal despite high acceptance of other maternal vaccines. Strengthening risk communication, addressing concerns regarding vaccine safety during pregnancy and lactation, and leveraging trusted healthcare workers as sources of information may improve vaccine confidence and uptake. Lessons learned from the COVID-19 pandemic should inform future maternal immunization programs and preparedness efforts for emerging infectious disease threats.

## Supplementary Information

Supplementary Material 1.

## Data Availability

The datasets upon which our findings are based belong to the project investigation team. For confidentiality reasons, the datasets are not publicly available. However, the datasets can be availed upon reasonable request from the corresponding author and with permission from the project team.

## Abbreviations

AIDS: Acquired Immune Deficiency Syndrome
aPR: Adjusted prevalence ratio
CI: Confidence Interval
COVID-19: Coronavirus disease 2019
HCWs: Health Care Workers
HIV: Human Immunodeficiency Virus
KNRH: Kawempe National Referral Hospital
LMICs: Low- and middle-income countries
MoH: Ministry of Health
PR: Prevalence Ratio
TT: Tetanus Toxoid
UNEPI: Uganda National Expanded Program on Immunization
WHO: World Health Organization
