# Supplementary material for: Acceptability and uptake of COVID-19 vaccines among pregnant and lactating women seeking services at a tertiary public hospital in Kampala, Uganda
Source: BMC Pregnancy Childbirth. 2026 Jul 25;26:1194. doi: 10.1186/s12884-026-09708-8 (PMC13685350; doi:10.1186/s12884-026-09708-8)
Supplement: Supplementary file 1 [file 12884_2026_9708_MOESM1_ESM.docx]

**Supplementary material 1. Responses to questions on contextual, individual and group influences, vaccination specific issues among study participants**

| **Variable/question, n=424** | **Frequency** | **Percentage** |
| --- | --- | --- |
| **Health system/Facility** | | |
| **While at the health facility, did you EVER receive any information from the health workers on COVID-19 vaccination?** | | |
| No | 250 | 59.0 |
| Yes | 174 | 41.0 |
| **Did you EVER receive any information from the health workers on COVID-19 vaccination among pregnant/lactating women?** | | |
| No | 325 | 76.7 |
| Yes | 99 | 23.3 |
| **Did you EVER attend any health education programs or teachings on COVID-19 vaccination at the health facility?** |  |  |
| No | 292 | 68.9 |
| Yes | 132 | 31.1 |
| **Communication and media environment** | | |
| **Does participant own or regularly has access to any of these? Television, Radio, Mobile phone (smart), Internet, Newspapers** | | |
| No | 64 | 15.1 |
| Yes | 360 | 84.9 |
| **Which social media platforms do they regularly use?†** |  |  |
| None | 201 | 47.4 |
| Facebook | 164 | 38.9 |
| WhatsApp | 211 | 49.8 |
| Twitter | 88 | 20.8 |
| YouTube | 41 | 9.7 |
| Tik Tok | 55 | 13.0 |
| Snapchat | 24 | 5.7 |
| Others | 3 | 0.7 |
| **Do you obtain information related to COVID-19 vaccination on your social media? n=223** | | |
| No | 74 | 33.2 |
| Yes | 149 | 66.8 |
| **Do you share information related to COVID-19 vaccination on your social media? n=223** | | |
| No | 157 | 70.4 |
| Yes | 66 | 29.6 |
| **Who do you trust the MOST for information on COVID-19 vaccination?†** |  |  |
| Health workers including community health workers | 380 | 89.6 |
| Family, friends, relatives, neighbors | 34 | 8.0 |
| Media such as radio, TV, internet | 16 | 3.8 |
| Leaders including politicians and local leaders | 6 | 1.4 |
| Religious leaders | 21 | 4.9 |
| **Who do you trust the LEAST for information on COVID-19 vaccination?†** |  |  |
| Health workers including community health workers | 14 | 3.3 |
| Family, friends, relatives, neighbors | 82 | 19.3 |
| Media such as radio, TV, internet | 139 | 32.8 |
| Leaders including politicians and local leaders | 223 | 52.6 |
| Religious leaders | 32 | 7.6 |
| **Have any media reports such as TV, radios, internet, newspapers, made you reconsider your choice to receive the COVID-19 vaccine?** | | |
| No | 243 | 57.3 |
| Unsure | 128 | 30.2 |
| Yes | 53 | 12.5 |
| **Do you recall any vaccine that was positively debated in the media? such as TV, radios, internet, newspapers** | | |
| No | 216 | 50.9 |
| Unsure | 170 | 40.1 |
| Yes | 38 | 9.0 |
| **Which vaccine do you recall being positively debated in the media?† n=38** | |  |
| Tetanus toxoid | 8 | 21.1 |
| Rabies | 1 | 2.6 |
| Typhoid | 1 | 2.6 |
| Yellow fever | 2 | 5.3 |
| Meningitis | 1 | 2.6 |
| Hepatitis b | 8 | 21.1 |
| Human Papilloma Virus (HPV) | 7 | 18.4 |
| Polio | 1 | 2.6 |
| COVID-19 | 19 | 50.0 |
| **Have you taken it or are you willing to take it? n=38** |  |  |
| No | 24 | 63.2 |
| Unsure | - |  |
| Yes | 14 | 36.8 |
| **Do you believe some media reports on people dying following COVID-19 vaccination?** | | |
| No | 226 | 53.3 |
| Unsure | 90 | 21.2 |
| Yes | 108 | 25.5 |
| **Does this affect your willingness to get vaccinated? n=108** |  |  |
| No | 51 | 47.2 |
| Unsure | 3 | 2.8 |
| Yes | 54 | 50.0 |
| **Influential leaders, gate keepers and anti or pro-vaccination lobbies** | | |
| **Do leaders (local, religious and, political) in your community support COVID-19 vaccination among pregnant/lactating women** | | |
| No | 170 | 40.1 |
| Unsure | 170 | 40.1 |
| Yes | 84 | 19.8 |
| **If a leader in your community (local, religious and, political) advocates against the COVID-19 vaccine, would you consider NOT taking it**? | | |
| No | 261 | 61.6 |
| Unsure | 103 | 24.3 |
| Yes | 60 | 14.2 |
| **Has your religious leader ever advocated against taking the COVID-19 vaccine?** | |  |
| No | 328 | 77.4 |
| Unsure | 89 | 21.0 |
| Yes | 7 | 1.7 |
| **If so, did you take their advice? n=7** |  |  |
| No | 4 | 57.1 |
| Yes | 3 | 42.9 |
| **Historical influences** | | |
| **Do you remember any events in the past that would discourage you from receiving the COVID-19 vaccine?** | | |
| No | 238 | 56.1 |
| Unsure | 147 | 34.7 |
| Yes | 39 | 9.2 |
| **If so, mention just one event? (one most important event), n=39** | |  |
| Rumors of people dying after COVID-19 vaccination | 15 | 38.5 |
| Experienced severe adverse events after COVID-19 vaccination (pain at injection site, fever, headache, body weakness) | 21 | 53.8 |
| Someone died after COVID-19 vaccination | 3 | 7.7 |
| **Has your community in the past ever refused to take any vaccine?** |  |  |
| No | 324 | 76.4 |
| Unsure | 96 | 22.6 |
| Yes | 4 | 0.9 |
| **If so why, did your community in the past ever refused to take any vaccine? (one MOST important reason), n=4** | | |
| Rumors that vaccine was for animals not humans | 1 | 25.0 |
| The vaccine kills and brings infertility | 1 | 25.0 |
| The vaccine quality was faulty, contents could have been changed at the health facilities | 2 | 50.0 |
| **Which vaccine** **did your community refuse to take? n=4** |  |  |
| COVID-19 | 4 | 100 |
| **Religion/culture/gender/socioeconomic** | | |
| **Do you know anyone who hasn’t taken the COVID-19 vaccine due to religious reasons**? |  |  |
| No | 271 | 63.9 |
| Unsure | 131 | 30.9 |
| Yes | 22 | 5.2 |
| **Do you know anyone who hasn’t taken the COVID-19 vaccine due to cultural/tribal/ethnicity reasons?** | | |
| No | 326 | 76.9 |
| Unsure | 87 | 20.5 |
| Yes | 11 | 2.6 |
| **Do you agree with them not taking the COVID-19 vaccine due to religious/cultural/tribal/ethnicity reasons? n=29** |  |  |
| No | 19 | 65.5 |
| Unsure | 6 | 20.7 |
| Yes | 4 | 13.8 |
| **Do you think they are risking their health by not taking the COVID-19 vaccine due to religious/cultural/tribal/ethnicity reasons? n=29** | | |
| No | 10 | 34.5 |
| Unsure | 8 | 27.6 |
| Yes | 11 | 37.9 |
| **Does your religion recommend COVID-19 vaccine?** |  |  |
| No | 2 | 0.5 |
| Unsure | 25 | 5.9 |
| Yes | 397 | 93.6 |
| **Does your culture/tribe/ethnicity recommend COVID-19 vaccine?** |  |  |
| No | 1 | 0.2 |
| Unsure | 69 | 16.3 |
| Yes | 354 | 83.5 |
| **If your religion/culture/tribe/ethnicity, does not encourage vaccination, for what reasons? (ONE most important reason), n=2** | | |
| The never preach or encourage about vaccination | 1 | 50.0 |
| They do not give us the reasons | 1 | 50.0 |
| **Does your religion recommend other vaccines?** |  |  |
| No | 1 | 0.2 |
| Unsure | 23 | 5.4 |
| Yes | 400 | 94.3 |
| **Does your culture/tribe/ethnicity recommend other vaccines**? |  |  |
| No | 14 | 3.3 |
| Unsure | 98 | 23.1 |
| Yes | 312 | 73.6 |
| **If no, which vaccines, are NOT recommended by your culture or religion†, n=14** | |  |
| Rabies | 1 | 7.1 |
| Tetanus Toxoid (TT) | 12 | 85.7 |
| **Would you refuse the COVID-19 vaccine if the vaccinator was from another religion than yourself?** | | |
| No | 265 | 62.5 |
| Unsure | 106 | 25.0 |
| Yes | 53 | 12.5 |
| **Would you refuse the COVID-19 vaccine if the vaccinator was from another culture/tribe/ethnicity than yourself?** | | |
| No | 328 | 77.4 |
| Unsure | 71 | 16.7 |
| Yes | 25 | 5.9 |
| **Politics, policies (mandates)** | | |
| **Do you trust that the government in making decisions in your best interest by providing the COVID-19 vaccine?** | | |
| No | 112 | 26.4 |
| Unsure | 171 | 40.3 |
| Yes | 141 | 33.3 |
| **Did you ever DISAGREE with any of the choice of COVID-19 vaccines that were provided?** | | |
| No | 279 | 65.8 |
| Unsure | 141 | 33.3 |
| Yes | 4 | 0.9 |
| **If so, which COVID-19 vaccines did you disagree with?† n=4** |  |  |
| Astra Zeneca | 3 | 75.0 |
| Pfizer | 3 | 75.0 |
| Moderna | 3 | 75.0 |
| Johnson & Johnson | 3 | 75.0 |
| **Why did you disagree with this COVID-19 vaccine choice? n=4** |  |  |
| Too many doses | 1 | 25.0 |
| Most people I know who received Johnson got serious side effects | 1 | 25.0 |
| Not safe for pregnant women | 1 | 25.0 |
| Don’t like injectable vaccines | 1 | 25.0 |
| **I am convinced that the government has purchased high quality vaccines for COVID-19** | | |
| No | 148 | 34.9 |
| Unsure | 167 | 39.4 |
| Yes | 109 | 25.7 |
| **Did you EVER have an impression that the government or health workers were not providing the good quality COVID-19 vaccines?** | | |
| No | 253 | 59.7 |
| Unsure | 146 | 34.4 |
| Yes | 25 | 5.9 |
| **I only took the COVID-19 vaccine because my employer or someone else mandated me to, n=219** | | |
| No | 212 | 96.8 |
| Yes | 7 | 3.2 |
| **If they had NOT mandated me to take it, I would not have had it, n=7** |  |  |
| No | 4 | 57.1 |
| Unsure | - |  |
| Yes | 3 | 42.9 |
| **Geographic barriers** | | |
| **Has distance from health facility EVER prevented you from getting the COVID-19 vaccine?** | | |
| Has never attempted to get the vaccine | 130 | 30.7 |
| No | 206 | 48.6 |
| Unsure | 71 | 16.7 |
| Yes | 17 | 4.0 |
| **Has timing of vaccination (schedule) at the health facility EVER prevented you from getting the COVID-19 vaccine?** |  |  |
| Has never attempted to get the vaccine | 149 | 35.1 |
| No | 220 | 51.9 |
| Unsure | 33 | 7.8 |
| Yes | 22 | 5.2 |
| **Have waiting lines at health facility EVER prevented you from getting the COVID-19 vaccine?** | | |
| Has never attempted to get the vaccine | 146 | 34.4 |
| No | 219 | 51.6 |
| Unsure | 34 | 8.0 |
| Yes | 25 | 5.9 |
| **Have clinic costs/fees EVER prevented you from getting the COVID-19 vaccine?** | |  |
| Has never attempted to get the vaccine | 146 | 34.4 |
| No | 213 | 50.2 |
| Unsure | 44 | 10.4 |
| Yes | 21 | 4.9 |
| **The effort and time of waiting at the health facility is NOT worth for receiving the COVID-19 vaccine** | | |
| No | 281 | 66.3 |
| Unsure | 120 | 28.3 |
| Yes | 23 | 5.4 |
| **The cost of travelling to the health facility is NOT worth for receiving the COVID-19 vaccine** |  |  |
| No | 282 | 66.5 |
| Unsure | 125 | 29.5 |
| Yes | 17 | 4.0 |
| **The time, effort, cost, travelling and waiting at the health facility is not worth for receiving the COVID-19 vaccine** | | |
| No | 272 | 64.1 |
| Unsure | 136 | 32.1 |
| Yes | 16 | 3.8 |
| **What is the maximum amount of time you would be willing to spend in waiting at the health facility to get the COVID-19 vaccine?** | | |
| 5-20 minutes | 149 | 35.1 |
| >20-30 minutes | 74 | 17.5 |
| >30-60 minutes | 176 | 41.5 |
| >60-120 minutes | 16 | 3.8 |
| Not sure | 8 | 1.9 |
| Any time | 1 | 0.2 |
| **If you have to wait more than one hour at the facility to get the COVID-19 vaccine, is it worth travelling for?** | | |
| No | 175 | 41.3 |
| Unsure | 116 | 27.4 |
| Yes | 133 | 31.4 |
| **Do you have a lifestyle that prevents you from getting vaccines (moves or travels a lot throughout the year, or stays in multiple places for limited periods of time)?** | | |
| No | 390 | 92.0 |
| Yes | 34 | 8.0 |
| **Pharmaceutical industry** | | |
| **Do you believe the COVID-19 vaccine producers are interested in your health?** | |  |
| No | 121 | 28.5 |
| Unsure | 181 | 42.7 |
| Yes | 122 | 28.8 |
| **Do you think the government was pushed by lobbyists or industry to recommend COVID-19 vaccination?** | | |
| No | 263 | 62.0 |
| Unsure | 148 | 34.9 |
| Yes | 13 | 3.1 |
| **Do you trust that pharmaceutical companies are providing safe and effective COVID-19 vaccines?** |  |  |
| No | 164 | 38.7 |
| Unsure | 150 | 35.4 |
| Yes | 110 | 25.9 |
| **Do you trust that pharmaceutical companies are providing safe and effective COVID-19 vaccines for pregnant and lactating women?** | | |
| No | 195 | 46.0 |
| Unsure | 116 | 27.4 |
| Yes | 113 | 26.6 |
| **Section 3: Individual and group influences (influences arising from personal perception of the vaccine or influences of the social/peer environment)** | | |
| **Experience with past vaccinations** |  |  |
| **Have you ever refused a vaccine?** |  |  |
| No | 399 | 94.1 |
| Yes | 25 | 5.9 |
| **Which vaccine did you refuse? n=25** |  |  |
| Don’t know the name | 1 | 4 |
| Ebola | 1 | 4 |
| COVID-19 | 21 | 84 |
| **For what reason did you refuse the vaccines? n=25** |  |  |
| Due to fear of side effects | 7 | 28.0 |
| Had experienced bad effects with the vaccine | 1 | 4.0 |
| Don’t trust the vaccine | 4 | 16.0 |
| Many myths about the vaccine | 3 | 12.0 |
| Not safe for pregnant women | 6 | 24.0 |
| Too many deaths from the vaccines | 1 | 4.0 |
| Lack of information about the vaccine | 2 | 8.0 |
| Distance to health facilities | 1 | 4.0 |
| **Do you think most pregnant or lactating women tolerate vaccines very well?** |  |  |
| No | 199 | 46.9 |
| Unsure | 183 | 43.2 |
| Yes | 42 | 9.9 |
| **Have you or someone you know ever had a bad reaction to vaccine that made you change your mind about vaccines?** |  |  |
| No | 283 | 66.8 |
| Unsure | 115 | 27.1 |
| Yes | 26 | 6.1 |
| **Do you know any pregnant or lactating woman who had a bad reaction to COVID-19 vaccine?** | | |
| No | 327 | 77.1 |
| Unsure | 92 | 21.7 |
| Yes | 5 | 1.2 |
| **Did that make you change your mind about the vaccines? n=5** |  |  |
| No | 3 | 60.0 |
| Yes | 2 | 40.0 |
| **Which bad reaction was it? n=5** |  |  |
| Severe allergic reaction | 2 | 40.0 |
| Neurological side effects such as seizures | 1 | 20.0 |
| Headache | 1 | 20.0 |
| Fatigue or loss of appetite | 1 | 20.0 |
| Fever, chills | 2 | 40.0 |
| Miscarriage | 1 | 20.0 |
| **Past vaccinations** | | |
| **Do you know anyone who died after COVID-19 vaccination?** |  |  |
| No | 231 | 54.5 |
| Unsure | 180 | 42.4 |
| Yes | 13 | 3.1 |
| **Did that change your mind on getting the vaccine? n=13** |  |  |
| No | 6 | 46.2 |
| Unsure | - |  |
| Yes | 7 | 53.8 |
| **Do you know any pregnant or lactating woman who died after COVID-19 vaccination?** | | |
| No | 346 | 81.6 |
| Unsure | 77 | 18.2 |
| Yes | 1 | 0.2 |
| **Did that change your mind on getting the vaccine? n=1** |  |  |
| No | 1 | 100 |
| Unsure | - |  |
| Yes | - |  |
| **Do experiences with pain after vaccinations prevent you from taking the COVID-19 vaccine?** | | |
| No | 328 | 77.4 |
| Unsure | 73 | 17.2 |
| Yes | 23 | 5.4 |
| **Beliefs attitudes about Health and prevention** | | |
| **Do you think the COVID-19 vaccine overloads the immune system among pregnant or lactating women?** |  |  |
| No | 146 | 34.4 |
| Unsure | 250 | 59.0 |
| Yes | 28 | 6.6 |
| **Do you believe there are other ways COVID-19 would be prevented instead of the vaccine?** | | |
| No | 212 | 50.0 |
| Unsure | 66 | 15.6 |
| Yes | 146 | 34.4 |
| **Knowledge/awareness** | | |
| **Do you feel you know which type/choice of COVID-19 vaccine to get?** |  |  |
| No | 367 | 86.6 |
| Yes | 57 | 13.4 |
| **Does the vaccinator provide enough information for COVID-19 vaccination among pregnant or lactating women?** |  |  |
| No | 216 | 50.9 |
| Unsure | 169 | 39.9 |
| Yes | 39 | 9.2 |
| **Do you inform yourself about COVID-19 vaccination among pregnant/lactating women?** |  |  |
| No | 368 | 86.8 |
| Yes | 56 | 13.2 |
| **Do you feel you have enough information about COVID-19 vaccines and their safety in pregnancy or lactating women?** | | |
| No | 222 | 52.4 |
| Unsure | 158 | 37.3 |
| Yes | 44 | 10.4 |
| **Do you prefer to receive more information about COVID-19 vaccination among pregnant/lactating women from the health facility?** | | |
| No | 161 | 38.0 |
| Unsure | 138 | 32.5 |
| Yes | 125 | 29.5 |
| **If so, would this make you choose to take the vaccine? n=125** |  |  |
| No | 11 | 8.8 |
| Unsure | 21 | 16.8 |
| Yes | 93 | 74.4 |
| **Have health care providers ever given all the information you need on COVID-19 vaccination?** | | |
| No | 202 | 47.6 |
| Unsure | 181 | 42.7 |
| Yes | 41 | 9.7 |
| **Are some vaccines more important than others during pregnancy or lactation?** | |  |
| No | 181 | 42.7 |
| Unsure | 215 | 50.7 |
| Yes | 28 | 6.6 |
| **If so, which vaccines are more important?† n=28** |  |  |
| Tetanus toxoid | 26 | 92.8 |
| Rabies | 3 | 10.7 |
| Typhoid | 3 | 10.7 |
| Yellow fever | 3 | 10.7 |
| Meningitis | 1 | 3.6 |
| Hepatitis B | 13 | 46.4 |
| **Health system and providers: Trust and personal experience** | | |
| **Do you think Information of COVID-19 vaccine side effects has been discussed openly by the public health authorities?** | | |
| No | 132 | 31.1 |
| Unsure | 222 | 52.4 |
| Yes | 70 | 16.5 |
| **Have you felt like health care professionals, government and local authorities are pushing you into COVID-19 vaccination, yet you do not fully support it?** | | |
| No | 282 | 66.5 |
| Unsure | 122 | 28.8 |
| Yes | 20 | 4.7 |
| **If so, why don't you support the COVID-19 vaccine? n=20** |  |  |
| Fear of side effects | 3 | 15.0 |
| They don’t discuss the side effects of the vaccine or teach the community | 5 | 25.0 |
| Not safe for pregnant women | 4 | 20.0 |
| We believe people died after getting the vaccine | 2 | 10.0 |
| Vaccine is just not safe for everyone | 4 | 20.0 |
| They used force to push us to get the vaccine | 2 | 10.0 |
| **Does having the same health care provider on each of your appointment make you more likely to have the COVID-19 vaccine?** | | |
| No | 237 | 55.9 |
| Unsure | 145 | 34.2 |
| Yes | 42 | 9.9 |
| **Would you discuss any concerns on the COVID-19 vaccine with your health care provider?** | | |
| No | 207 | 48.8 |
| Unsure | 139 | 32.8 |
| Yes | 78 | 18.4 |
| **Would you trust the information the health care provider gives you about the COVID-19 vaccine?** |  |  |
| No | 145 | 34.2 |
| Unsure | 139 | 32.8 |
| Yes | 140 | 33.0 |
| **Do you think that the health care provider cares about what is best for you?** | |  |
| No | 165 | 38.9 |
| Unsure | 111 | 26.2 |
| Yes | 148 | 34.9 |
| **Risk/benefit (perceived, heuristic)** | | |
| **How concerned are you that any of the COVID-19 vaccines may not be safe for pregnant or lactating women?** | | |
| Not concerned | 172 | 40.6 |
| Very concerned | 252 | 59.4 |
| **Do you think the COVID-19 vaccine will be needed even when the COVID-19 epidemic ends?** | | |
| No | 153 | 36.1 |
| Unsure | 149 | 35.1 |
| Yes | 122 | 28.7 |
| **How concerned are you that you may have a bad reaction after a COVID-19 shot? (If you had been vaccinated), n=219** | | |
| Not concerned | 160 | 73.1 |
| Very concerned | 59 | 26.9 |
| **If you had a bad reaction after the first COVID-19 jab, you would NOT return for the second jab?** | | |
| No | 272 | 64.2 |
| Unsure | 115 | 27.1 |
| Yes | 37 | 8.7 |
| **COVID-19 cases or deaths were NOT common where I live, that is why I am not willing to take the vaccine or not vaccinated, n=205** | | |
| No | 148 | 72.2 |
| Unsure | 45 | 21.9 |
| Yes | 12 | 5.9 |
| **Immunization as a social Norm vs. Not needed/harmful** | | |
| **I agree, it’s important for all pregnant or lactating women to get the COVID-19 vaccine** | | |
| No | 162 | 38.2 |
| Unsure | 219 | 51.7 |
| Yes | 43 | 10.1 |
| **Do you believe that all pregnant lactating women should get all recommended vaccines?** | | |
| No | 37 | 8.7 |
| Unsure | 310 | 73.1 |
| Yes | 77 | 18.2 |
| **If no, why do you think they should NOT get vaccinated? n=37** |  |  |
| They have weakened immunity; the vaccine is a risk | 3 | 8.1 |
| Not safe for the baby/pregnancy | 17 | 45.9 |
| New vaccines are not to be trusted | 3 | 8.1 |
| There are other ways to avoid the diseases instead of vaccines that have bad effects | 3 | 8.1 |
| Even those who are vaccinated could still get the disease and die | 2 | 5.4 |
| Pregnancy ends, they can get the vaccines after | 3 | 8.1 |
| They will experience very bad side effects | 6 | 16.2 |
| **Do you think it is important to get the COVID-19 vaccine in order to prevent those that are unable to access it from getting the disease?** | | |
| No | 10 | 2.3 |
| Unsure | 306 | 72.2 |
| Yes | 108 | 25.5 |
| **If no, why do you think it is NOT important? n=10** |  |  |
| There are many other ways to prevent people from getting COVID-19 | 5 | 50.0 |
| People can build natural immunity | 3 | 30.0 |
| Even the vaccinated can get COVID-19 | 2 | 20.0 |
| **Have the other pregnant or lactating women, in your community or circle of friends received the COVID-19 vaccine?** | | |
| No | 21 | 4.9 |
| Unsure | 367 | 86.6 |
| Yes | 36 | 8.5 |
| **If no, why have they not received the COVID-19 vaccine? n=21** |  |  |
| Fear of side effects | 14 | 66.7 |
| Lack of knowledge and information on COVID-19 vaccination for pregnant women | 6 | 28.6 |
| **Do you believe that if you get vaccinated against COVID-19, others are protected too?** | | |
| No | 106 | 25.0 |
| Unsure | 216 | 50.9 |
| Yes | 102 | 24.1 |
| **Are you worried that some of the pregnant or lactating women in the community are refusing to get the COVID-19 vaccines, and are potentially putting others at risk?** | | |
| No | 178 | 42.0 |
| Unsure | 188 | 44.3 |
| Yes | 58 | 13.7 |
| **Section 4: Vaccine/vaccination (specific issues directly related to COVID-19 vaccine or vaccination)** | | |
| **Risk/Benefit (scientific evidence)** |  |  |
| **Do you believe the COVID-19 vaccine is safe for you and the unborn/breast feeding baby?** | | |
| No | 153 | 36.1 |
| Unsure | 212 | 50.0 |
| Yes | 59 | 13.9 |
| **Do you think other recommended vaccines are safe for you and your unborn/breast feeding baby?** | | |
| No | 206 | 48.6 |
| Unsure | 122 | 28.8 |
| Yes | 96 | 22.6 |
| **Have you EVER experienced any bad reaction after any immunization?** |  |  |
| Has never been immunized | 13 | 3.1 |
| No | 235 | 55.4 |
| Unsure | 139 | 32.8 |
| Yes | 37 | 8.7 |
| **Did you get a bad reaction after the first COVID-19 dose? n=219** |  |  |
| No | 142 | 64.8 |
| Unsure | 56 | 25.6 |
| Yes | 21 | 9.6 |
| **If so, which bad reaction did you get after the first COVID-19 dose?** **n=21** |  |  |
| Cough | 1 | 4.8 |
| Fever | 18 | 85.7 |
| Arm heaviness | 3 | 14.3 |
| Headache | 1 | 4.8 |
| Nausea | 1 | 4.8 |
| Dizziness | 2 | 9.5 |
| Severe headache | 1 | 4.8 |
| Body swelling | 1 | 4.8 |
| General weakness | 1 | 4.8 |
| **Do you think some vaccines are much safer than the COVID-19 vaccine for pregnant and lactating women?** | | |
| No | 185 | 43.6 |
| Unsure | 204 | 48.1 |
| Yes | 35 | 8.3 |
| **If so, which vaccines are much SAFER for them?†** **n=35** |  |  |
| Tetanus toxoid | 34 | 97.1 |
| Rabies | 4 | 11.4 |
| Typhoid | 2 | 5.7 |
| Yellow fever | 3 | 8.6 |
| Meningitis | 1 | 2.8 |
| Hepatitis B | 10 | 28.6 |
| **And why are they much safer? n=35** |  |  |
| Pregnant women have always been taking them for years/are not new | 22 | 62.9 |
| You experience no side affects | 8 | 22.9 |
| They protect the mother and baby from tetanus | 5 | 14.3 |
| **Before receiving any vaccine, did health care providers give you information on the side effects?** | | |
| Does not remember | 6 | 1.4 |
| Has never received any vaccines | 18 | 4.3 |
| No | 182 | 42.9 |
| Unsure | 140 | 33.0 |
| Yes | 78 | 18.4 |
| **Introduction of a New vaccine or new Formulation** | | |
| **When the COVID-19 vaccine was introduced, did you rather wait and saw what other people were doing before getting it?** | | |
| No | 160 | 37.7 |
| Unsure | 143 | 33.7 |
| Yes | 121 | 28.5 |
| **Do you consider COVID-19 vaccine to be safe for pregnancy and lactation?** | |  |
| No | 212 | 50.0 |
| Unsure | 169 | 39.9 |
| Yes | 43 | 10.1 |
| **I also consider …………………. Vaccines to be SAFE during pregnancy and lactation†** | |  |
| Tetanus toxoid | 397 | 93.6 |
| Rabies | 22 | 5.2 |
| Typhoid | 19 | 4.5 |
| Yellow fever | 22 | 5.2 |
| Meningitis | 6 | 1.4 |
| Hepatitis B | 57 | 13.4 |
| **Do you think that you are at risk of getting COVID-19 disease if NOT vaccinated?** | |  |
| No | 173 | 40.8 |
| Unsure | 131 | 30.9 |
| Yes | 120 | 28.3 |
| **Do you think COVID-19 vaccines was NOT passed through enough rigorous standards like the other vaccines?** | | |
| No | 188 | 44.3 |
| Unsure | 216 | 50.9 |
| Yes | 20 | 4.7 |
| **Do you think COVID-19 was not passed through enough rigorous standards among pregnant and lactating women like the other vaccines or drugs that are safe for these groups** | | |
| No | 210 | 49.5 |
| Unsure | 189 | 44.6 |
| Yes | 25 | 5.9 |
| **Have you ever hesitated to receive a new vaccine?** |  |  |
| No | 402 | 94.8 |
| Yes | 22 | 5.2 |
| **Which new vaccine have you ever hesitated to receive? n=22** |  |  |
| Ebola | 1 | 4.5 |
| COVID-19 | 21 | 95.5 |
| **Mode of administration** | | |
| **Which mode of vaccination (injection or oral) do you PREFER? (For any vaccine)** | |  |
| Injections or oral | 227 | 53.5 |
| Injections only | 142 | 33.5 |
| Oral only | 55 | 13.0 |
| **Does the fear for pain or needles make you hesitate to take the COVID-19 vaccine?** | |  |
| No | 269 | 63.4 |
| Unsure | 131 | 30.9 |
| Yes | 24 | 5.7 |
| **Has pain after immunization ever made you regret your decision to vaccinate?** | |  |
| No | 288 | 67.9 |
| Unsure | 117 | 27.6 |
| Yes | 19 | 4.5 |
| **If so, which vaccine made you regret your decision to vaccinate? n=19** |  |  |
| Don’t know the name | 4 | 21.1 |
| Rabies | 1 | 5.3 |
| Tetanus Toxoid | 12 | 63.2 |
| COVID-19 | 2 | 10.5 |
| **Would you be willing to get additional doses of COVID-19 vaccine if there was NO pain from needles?** | | |
| No | 213 | 50.2 |
| Unsure | 142 | 33.5 |
| Yes | 69 | 16.3 |
| **Do you trust your health care provider to safely administer the COVID-19 vaccine?** | |  |
| No | 164 | 38.7 |
| Unsure | 93 | 21.9 |
| Yes | 167 | 39.4 |
| **Design of vaccination program/Mode of delivery** | | |
| **Is the COVID-19 vaccination process welcoming?** |  |  |
| Has not been to COVID-19 vaccination site | 131 | 30.9 |
| No | 42 | 9.9 |
| Unsure | 88 | 20.7 |
| Yes | 163 | 38.4 |
| **Would you rather receive the COVID-19 vaccine as quickly and convenient as possible OR would you rather spend much more time on consultation?** | | |
| No (would you rather spend much more time on consultation) | 405 | 95.5 |
| Unsure | 16 | 3.8 |
| Yes (receive the COVID-19 vaccine as quickly and convenient as possible) | 3 | 0.7 |
| **What would you prefer about receiving the COVID-19 vaccine? †** |  |  |
| Door to door | 60 | 14.2 |
| Health center | 235 | 55.4 |
| Mass campaign in community | 68 | 16.0 |
| Personal doctor | 26 | 6.1 |
| From schools, institution-based programs | 122 | 28.8 |
| **Would you rather get vaccinated at the workplace or place of business?** |  |  |
| Has no workplace | 106 | 25.0 |
| No | 149 | 35.1 |
| Unsure | 140 | 33.0 |
| Yes | 29 | 6.8 |
| **If yes? Why would you rather get vaccinated at the workplace or place of business? n=29** | | |
| Saves time | 17 | 58.6 |
| Saves money on transport to health facility | 3 | 10.3 |
| It is convenient | 7 | 24.1 |
| It is comfortable | 2 | 6.9 |
| **Would you refrain/STAY AWAY/REFUSE a mass COVID-19 vaccine campaign in the community?** | | |
| No | 264 | 62.3 |
| Unsure | 159 | 37.5 |
| Yes | 1 | 0.2 |
| **If yes, WHY would you refrain/STAY AWAY/REFUSE a mass COVID-19 vaccine campaign in the community?** **n=1** | | |
| They don't teach before vaccination | 1 | 100 |
|  |  |  |
| **Reliability and/or Source of vaccine Supply** | | |
| **Do you feel confident that the health facility will have the COVID-19 vaccine when you need it?** | | |
| No | 169 | 39.8 |
| Unsure | 167 | 39.4 |
| Yes | 88 | 20.8 |
| **Did you ever go to the health center or vaccination post in community to get the COVID-19 vaccine and NEVER received it?** | | |
| No | 281 | 66.3 |
| Unsure | 134 | 31.6 |
| Yes | 9 | 2.1 |
| **If yes, when did you go to the health center or vaccination post in community to get the COVID-19 vaccine**? **n=9** |  |  |
| 2023 | 1 | 11.1 |
| 2022 | 5 | 55.6 |
| 2021 | 3 | 33.3 |
| **If yes, why did you not receive it? n=9** |  |  |
| Stock outs | 6 | 66.7 |
| Had no ID card | 1 | 11.1 |
| Health worker tired | 1 | 11.1 |
| Long lines | 1 | 11.1 |
| **Did you get it eventually? n=9** |  |  |
| Yes | 0 | 0 |
| No | 9 | 100 |
| **Did the health provider REFUSE to give the COVID-19 vaccine because there were too few people to start the batch?** | | |
| Has not been to the health facility | 130 | 30.7 |
| No | 192 | 45.3 |
| Unsure | 96 | 22.6 |
| Yes | 6 | 1.4 |
| **Are you concerned about where the COVID-19 vaccine is manufactured?** |  |  |
| No | 298 | 70.3 |
| Unsure | 123 | 29.0 |
| Yes | 3 | 0.7 |
| **Have you ever declined any vaccines because of the country where it was manufactured?** | | |
| No | 339 | 79.9 |
| Unsure | 85 | 20.1 |
| Yes | 0 | 0 |
| **Vaccination schedule** | | |
| **Are there any other vaccines that you have had trouble accessing due to their schedules at the facility?** | | |
| I have never tried to access vaccines | 20 | 4.7 |
| No | 225 | 53.1 |
| Unsure | 173 | 40.8 |
| Yes | 6 | 1.4 |
| **Would you follow the recommended shot schedule for COVID-19 vaccine?** **(8 weeks after the first dose)** | | |
| No | 237 | 55.9 |
| Unsure | 95 | 22.4 |
| Yes | 92 | 21.7 |
| **Is it better for pregnant and lactating women, to get FEWER vaccines at a time?** | |  |
| No | 177 | 41.8 |
| Unsure | 224 | 52.8 |
| Yes | 23 | 5.4 |
| **Do you think Pregnant/lactating women can wait to get the second shot of COVID-19?** | | |
| No | 380 | 89.6 |
| Yes | 44 | 10.4 |
| **If yes, why do you think they can wait to get the second shot of COVID-19?** **n=44** | | |
| For safety of the baby/pregnancy | 8 | 18.2 |
| It may change their immunity | 2 | 4.5 |
| They should wait to seek for information and knowledge on the vaccine | 15 | 34.1 |
| The vaccine is too strong, they should wait | 5 | 11.4 |
| To allow the bodies to get used/recover from previous dose | 4 | 9.1 |
| To monitor if they might get a problem from the first dose | 2 | 4.5 |
| They should wait at least until the give birth | 8 | 18.2 |
| **Costs** |  |  |
| **If the COVID-19 vaccine was offered at a cost, would you still get it?** |  |  |
| No | 207 | 48.8 |
| Unsure | 167 | 39.4 |
| Yes | 50 | 11.8 |
| **If yes, at what cost? (Ugandan Shillings)** **n=50** |  |  |
| <6,000 | 12 | 24.0 |
| 6,000-10,000 | 18 | 36.0 |
| 11,000-20,000 | 4 | 8.0 |
| 21,000-50,000 | 12 | 24.0 |
| >50,000 | 4 | 8.0 |
| **Do you avoid/refrain from getting other vaccines that are offered at a cost?** | |  |
| No | 296 | 69.8 |
| Unsure | 119 | 28.1 |
| Yes | 9 | 2.1 |
| **If yes, which ones do you avoid/refrain from getting (other vaccines that are offered at a cost)?†** | | |
| Tetanus toxoid | 6 | 66.7 |
| Rabies | 2 | 22.2 |
| Typhoid | 1 | 11.1 |
| Yellow fever | 2 | 22.2 |
| Meningitis | 2 | 22.2 |
| Hepatitis B | 4 | 44.4 |
| **Does the cost of any vaccine prevent you from getting it even when you felt you needed it?** | | |
| No | 297 | 70.1 |
| Unsure | 113 | 26.7 |
| Yes | 14 | 3.3 |
| **Which vaccines do you trust more?** |  |  |
| The ones free of charge | 383 | 90.3 |
| The ones offered at a cost | 41 | 9.7 |
| **Would you pay for additional vaccines? Other than those offered free of charge at your health facility**? | | |
| No | 256 | 60.4 |
| Unsure | 108 | 25.5 |
| Yes | 60 | 14.1 |
| **Would you mind taking time off from work or from your business to get the COVID-19 vaccine?** | | |
| No | 227 | 53.5 |
| Unsure | 150 | 35.4 |
| Yes | 47 | 11.1 |
| **Which vaccines would you be willing to pay for? Including those offered free of charge†** | | |
| Tetanus toxoid | 288 | 67.9 |
| Rabies | 37 | 8.7 |
| Typhoid | 13 | 3.1 |
| Yellow fever | 43 | 10.1 |
| Meningitis | 2 | 0.5 |
| Hepatitis B | 53 | 12.5 |
| **Role of healthcare professionals** | | |
| **Did health care providers EVER treat you without respect (with regards to your appearance, education or cultural background)?** | | |
| No | 280 | 66.0 |
| Unsure | 138 | 32.6 |
| Yes | 6 | 1.4 |
| **Did this EVER stop you from visiting the health facility when they treated you without respect?** **n=6** | | |
| No | 2 | 33.3 |
| Unsure | 2 | 33.3 |
| Yes | 2 | 33.3 |
| **Would you choose a specific health care worker to give you the COVID-19 vaccine?** | |  |
| No | 313 | 73.8 |
| Unsure | 99 | 23.4 |
| Yes | 12 | 2.8 |
| **Would you mind if a specific health worker is not available at the time when you need the COVID-19 vaccine?** | | |
| No | 289 | 68.2 |
| Unsure | 122 | 28.7 |
| Yes | 13 | 3.1 |
| **Has any health care worker advised you against taking the COVID-19 vaccine?** | |  |
| No | 294 | 69.3 |
| Unsure | 129 | 30.4 |
| Yes | 1 | 0.2 |
| **If yes, why did the health care worker advise you against taking the COVID-19 vaccine?** **n=1** | | |
| Safety issues | 1 | 100 |
| **Has any health care worker advised you against taking any other vaccines?** | |  |
| I have never talked to a health worker | 23 | 5.4 |
| No | 262 | 61.8 |
| Unsure | 138 | 32.5 |
| Yes | 1 | 0.2 |
| **Was a health care worker ever reluctant to give the COVID-19 vaccine when you requested?** | | |
| I have never requested for the COVDI-19 vaccine | 130 | 30.7 |
| No | 293 | 69.1 |
| Yes | 1 | 0.2 |
| **If yes, why was a health care worker ever reluctant to give the COVID-19 vaccine when you requested?** **n=1** | | |
| They were tired | 1 | 100 |
| **Do you trust door to door vaccination for COVID-19**? |  |  |
| No | 89 | 21.0 |
| Unsure | 42 | 9.9 |
| Yes | 293 | 69.1 |
| † Multiple responses were allowed; percentages may exceed 100%. | | |
